# The interaction of ABA and ROS in plant growth and stress resistances

**DOI:** 10.3389/fpls.2022.1050132

**Published:** 2022-11-24

**Authors:** Shenghui Li, Sha Liu, Qiong Zhang, Meixiang Cui, Min Zhao, Nanyang Li, Suna Wang, Ruigang Wu, Lin Zhang, Yunpeng Cao, Lihu Wang

**Affiliations:** ^1^ School of Landscape and Ecological Engineering, Hebei University of Engineering, Handan, China; ^2^ Institute of Pomology, Shandong Academy of Agricultural Sciences, Tai’an, China; ^3^ School of Basic Medical Sciences, Hubei University of Chinese Medicine, Wuhan, China; ^4^ Key Laboratory of Plant Germplasm Enhancement and Specialty Agriculture, Wuhan Botanical Garden, Chinese Academy of Sciences, Wuhan, China

**Keywords:** ABA, ROS, plant growth, resistances, signal regulation

## Abstract

The plant hormone ABA (abscisic acid) plays an extremely important role in plant growth and adaptive stress, including but are not limited to seed germination, stomatal closure, pathogen infection, drought and cold stresses. Reactive oxygen species (ROS) are response molecules widely produced by plant cells under biotic and abiotic stress conditions. The production of apoplast ROS is induced and regulated by ABA, and participates in the ABA signaling pathway and its regulated plant immune system. In this review, we summarize ABA and ROS in apoplast ROS production, plant response to biotic and abiotic stresses, plant growth regulation, ABA signal transduction, and the regulatory relationship between ABA and other plant hormones. In addition, we also discuss the effects of protein post-translational modifications on ABA and ROS related factors.

## Introduction

In nature, plants must adapt to their living environment to survive, which achieve the goal of individual growth and population reproduction, especially when plants are under stress conditions, including abiotic and biotic stresses. With the deepening of research, it has been found that ABA, as an important plant hormone. The main source of ABA is a *de novo* synthesis pathway that relies on carotenoids in higher plants (Nambara and Marion-Poll, 2005). ABA signal transduction begins with the ABA receptor perceiving the ABA signal, and then transmits the ABA signal downwards through a series of regulatory factors in the cell, forming a complex intracellular signal transduction network, and finally transformed into a visible physiological effect (Hauser et al., 2011). The regulatory network downstream of the ABA signal has protein phosphatase Type-2C Protein Phosphatase (PP2C) as the main node in the cell. In plants, PP2C is a serine/threonine protein phosphatase as a negative regulator in the ABA signaling pathway (Santiago et al., 2009; Umezawa et al., 2009). Studies have reported that PP2C functionally acquired mutants *abi1-1* and *abi2-1* are highly insensitive to ABA in seed germination and seedling growth (Gosti et al., 1999). In addition, the important regulatory factors downstream of ABA signal also include a large number of transcription factors that transmit and realize the gene functions regulated by ABA signal, such as ABI3, ABI4, ABI5, etc. these are all important transcription factors in ABA affecting seed germination and seedling morphology (Finkelstein et al., 2002).

In plants, ROS are stress-responsive substance, such as hydrogen peroxide (H_2_O_2_), superoxide anion (O_2_^-^), hydroxyl radical (OH^-^). In the process of aerobic metabolism, such as respiration and photosynthesis could inevitably lead to the production of various ROS in mitochondria, chloroplast and peroxisome, and a large amount of cellular ROS will also be caused in various biotic and abiotic stresses, and then leading to programmed cell death (PCD). A common feature of these different sources and types of ROS is that they cause oxidative damage to living substances such as protein, DNA, and lipid. Therefore, the balance of ROS in plants will also be strictly regulated. Plants have two ROS scavenging systems, namely the enzymatic scavenging system and the non-enzymatic scavenging system (Mittler, 2002; Mittler, 2017). The enzyme scavenging system is mainly through the reduction of enzymatic peroxides such as superoxide dismutase (SOD) and peroxidase (POD) to eliminate ROS, and the non-enzymatic scavenging system uses the ascorbate-glutathione cycle to eliminate ROS (Mittler, 2002). Interestingly, some studies found that there are many different mechanisms in plants that link ABA signaling with redox balance. Firstly, ABA induces the transcription of genes related to the ascorbate-glutathione cycle in the ROS scavenging system (Ghassemian et al., 2008). Secondly, the oxidation-decyclization from xanthine to zeaxanthin requires ascorbate oxidize to dehydroascorbate in ABA biosynthesis (Nambara and Marion-Poll, 2005; Hartung, 2010), which indirectly affects the level of ascorbate and the content of ROS (Ye et al., 2012), and also explains why ABA deficient mutants have lower ascorbate content and higher ROS enrichment (Ton et al., 2009).

ABA mediates the response of plants to a variety of environmental stresses, including abiotic stresses such as drought, salt, osmotic and cold stresses (Yoshida et al., 2014; Arif et al., 2018; Huang et al., 2018; Silva et al., 2018). Simultaneously, the degree of stress hormones response is also related to the adaptive capacity of plants to these adversity conditions (Nambara and Marion-Poll, 2005). Meanwhile, the role of ROS in plants which subjected to adversity stresses has been widely reported. Some studies believe that the accumulation of ROS is an adaptive response of plants under stress conditions (Suzuki et al., 2011), and it is not simply toxicity of metabolism. As a by-product, more and more evidences show that ROS also plays a role as a signaling molecule to regulate plant development and the expression of stress response genes, including encoding antioxidant enzyme genes, in order to regulate the production of H_2_O_2_ (Neill, 2002; Gechev et al., 2003; Choudhury et al., 2017; Mittler, 2017; Xue et al., 2020).

In addition, the function of ABA in plant-pathogen interactions has also been intensively studied (Ton et al., 2009; Cao et al., 2011). When facing multiple stresses, plants will initiate responses in the order of stresses or severity. In many cases though, ABA could promote the process of abiotic stress response to accelerate the initiation of defense responses to biotic stresses, such as pathogenic fungi (Robert-Seilaniantz et al., 2007; AbuQamar et al., 2017; Darma et al., 2019). In the early view, ROS would damage plant cell and accelerate PCD, as a signal molecule involved in the normal disease resistance process of plants, considered a universal early response substance to the infection of pathogenic microorganisms (Wong et al., 2008; Chi et al., 2009).

ABA not only promoted the shedding of plant organs, but also participates in the other plant growth and development processes, such as seed maturation, dormancy and germination, root growth and flower development (Leung and Giraudat, 1998; White et al., 2000; Finkelstein et al., 2002; Yoshida et al., 2006; Fujii et al., 2007). With the continuous progress of research, it has been found that under certain stress conditions, plants could generate a large amount of ROS to inhibit plant growth and development (Mittler, 2002; Agurla et al., 2018; Muhlemann et al., 2018), affect plant cell differentiation, root growth and stomata closure, etc. (Dietz et al., 2016; Simmons and Bergmann, 2016; Xu et al., 2017a).

At present, most researches concentrate on the role of ABA as a signal molecule in the response of plants to multiple environmental factors, and focus on the crosstalk between ABA and the response to biotic or abiotic stress in plants (Ma et al., 2009; Lee and Luan, 2012; Skubacz et al., 2016). ROS not only strengthens the cell wall through protein cross-linking to achieve antibacterial effect, but also could be used as a signal molecule to induce plant hypersensitivity (HY) and systemic acquired resistance (SAR) (Apel and Hirt, 2004; Xu et al., 2017b). In turn, plant pathogen associated molecular patterns, bacterial effector, signal transcription cascade, ion movement and protein kinase activation, etc., these biological processes will further promote the production of ROS. Meanwhile, another molecular mechanism has also been proposed to explain the inhibitory effect of ABA on ROS enrichment (Grant and Jones, 2009), that ABA may affect the stability of DELLA protein and induce the expression of genes which encoding ROS scavenging system related enzymes to inhibit the accumulation of ROS, among them, DELLA protein is one of the key regulators that integrate the plant hormones and environmental signals (Achard et al., 2008; Yang et al., 2012), indicating that there is a complex regulatory relationship between ABA and ROS.

## The roles of ABA and ROS in plant resistances

**Figure 1:**
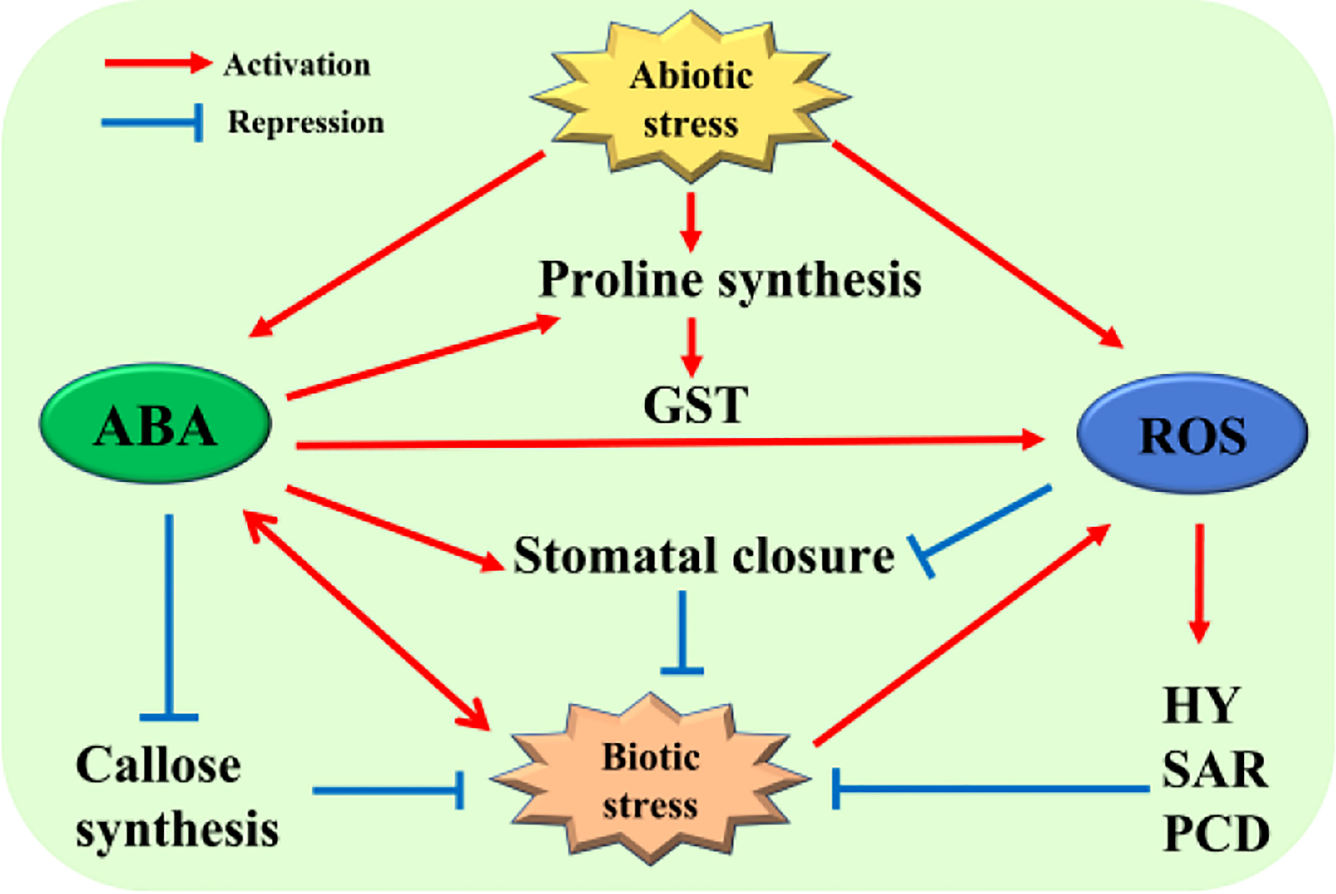
Regulatory network of the plant response to stress between ABA and ROS. Both biotic and abiotic stresses can cause the accumulation of ABA and ROS in plants, and ABA and ROS can also inhibit or adapt to external stress by regulating the content of some substances in plants or regulating the defense response of plants to maintain normal growth and development. For detailed explanation, please see text.

In adversities, such as low temperature, high light intensity, osmotic stress, etc., the content of plant endogenous hormones will change significantly and affect the physiological state of plants. Research reported that plant endogenous hormones have undergone great changes under low temperature stress, such as the decrease of gibberellin content and the sharp increase of ABA content (Shu et al., 2018). In the research on the changes of plant hormones under low temperature stress, which had more attention to ABA, showing that ABA is closely related to the cold resistance of plants (Huang et al., 2017; Agurla et al., 2018; Lv et al., 2018). It is generally believed that the ABA content of plant varieties with strong cold resistance is higher than that of varieties with weak cold resistance, and the ABA content is positively correlated with the cold resistance of plants (Huang et al., 2017; Rubio et al., 2019). Meanwhile, the rate of calvin cycle metabolism decreases under low temperature stress, which will lead to excessive consumption of photoreaction and accumulation of ROS in photosynthesis (Ensminger et al., 2006). Studies had suggested that ABA could improve oxidase activity and induce stomatal closure to reduce CO2 fixation, thereby inhibit the accumulation of ROS (Lim et al., 2015). However, a recent study has shown that OPEN STOMATA 1 (OST1), as an important kinase in the ABA signaling pathway, can phosphorylate the photosynthetic oxygen-producing protein PPD5 of chloroplasts in the photosynthesis to reduce the accumulation of ROS, promote stomatal opening, and lead to weaken the photo-protective mechanism of plants, that reflects the negative regulation effect of ABA in regulating stomata opening and drought resistance (Hong et al., 2020). In plants, proline is considered to be a molecular substance with multiple functions, many studies have shown that the enrichment of proline can promote the response of plants to different abiotic stresses (Haudecoeur et al., 2009; Szabados and Savouré, 2010; Kavi Kishor and Sreenivasulu, 2014). As a substance related to osmotic regulation, proline has been recognized as a protective molecule that protects plant cells from osmotic stress, and proline has the ability to increase the activity of antioxidant enzymes, implying its positive regulation of ROS scavenging activity function (Matysik et al., 2002). In addition, with the increase of ROS levels during different metabolic changes caused by abiotic stresses (Foyer and Noctor, 2005), ABA signaling and ABA-dependent proline enrichment have been shown as an important part of the tolerance signal network while the plants resist different stresses (Pastori and Foyer, 2002). Under osmotic and salt stresses, the activities of proline biosynthesis related enzymes pyrroline-5-carboxylate synthetase (P5CS) and P5C reductase (P5CR) (Hu et al., 1992) are activated by ABA (Verslues et al., 2007; Szabados and Savouré, 2010). Research finding that the reduction of ROS enrichment is accompanied by the increase of proline content in the *P5CS* over-expression transgenic tobacco (Hong et al., 2000; Siripornadulsil et al., 2002). Correspondingly, the decrease of proline level promotes the ROS enrichment and leads to oxidative damage of plant cells in the *p5cs1* insertion mutant (Szekely et al., 2008). Previous reports have also shown that salt stress-induced proline enrichment is dependent on the presence of ABA, which is consistent with the regulation of proline on redox, indicating that proline may act as an antioxidant in plants (Frank and James, 2006; Hoque et al., 2008). In addition, ABA also participates in ROS clearance process induced by salt stress in rice, however, ABA not only induces the expression of *OsP5CR* to generate proline through OsMADS25, but also induces the expression of *OsGST4* to generate glutathione S–transferase (GST), thereby promotes ROS clearance and enhances the salt tolerance of rice, simultaneously, this study also shows that auxin may play a very important role in the ABA regulation of ROS clearance (Xu et al., 2018) (**Figure 1**).

In the process of plant disease resistance and defense response, ABA exhibits dual characteristics in the process of pathogen infection and reproduction. For example, when plants are infected by the bacterial pathogen *Pseudomonas syringae pv. tomato* strain DC3000 (DC3000), ABA inhibits callose deposition and the expression of related genes induced by pathogen-associated molecular pattern (PAMP), that plays a negative regulatory role (de Torres Zabala et al., 2009). Meanwhile, when plant leaves are infected by bacterial pathogens, ABA will promote the closure of stomata to prevent the pathogens from spreading further in the plant (Melotto et al., 2006). In addition, ABA can promote the formation of callosum to block pathogenic bacteria continue to invade plants (Oide et al., 2013), that plays a positive regulatory role (**Figure 1**). The two-way regulation function is a hotspot on the interaction between plants and pathogens, and the kinetics of pathogen infection in the current research. When plants are infected by pathogens, that will induce the production of ROS and act as a signal molecule to activate plant hypersensitivity, which further starts systemic acquired resistance (SAR) (Apel and Hirt, 2004), or triggers off PCD to prevent further spread of the pathogens (Suzuki and Katano, 2018) (**Figure 1**). Interestingly, ABA can become the member of the infection strategy of pathogens in plants. For example, DC3000 can induce the enrichment of ABA and the expression of ABA signaling elements in *Arabidopsis thaliana*, this result is beneficial to the propagation of bacteria and the acceleration of the infection process (de Torres-Zabala et al., 2007). In addition, some pathogenic fungi, such as *Botrytis cinerea* and *Magnaporthe grisea*, directly promote the production of ABA to further accelerate the infection process and efficiency of pathogens (Audenaert et al., 2002; Cao et al., 2011).

## The relationship between ABA and ROS in plant growth and development

In plants, the root development is attributed to the elongation and growth of root cells, as well as the continuous division and differentiation of cells (Ramirez‐Parra et al., 2017), which is affected by the homeostasis of ROS in plants (Tsukagoshi et al., 2010). As an important signal molecule, ROS affects many aspects of plant growth and development, such as cell cycle, PCD, hormone signaling, and plant response to environmental stress (Xie et al., 2014; You et al., 2014; Leng et al., 2017; Tognetti et al., 2017), if the balance of ROS is disturbed, the growth and development of plants will be affected (Zhang et al., 2014). As an important hormone that regulates plant metabolism, ABA also plays an important role in the growth and development of plant roots. In *Arabidopsis*, the ABA hypersensitive mutant *abo6* accumulates a high concentration of ROS in the root tip, which slows down the growth rate of the root tip and reduced the activity of the root meristem (He et al., 2012; Yang et al., 2014). However, the exogenous application of GSH can partially restore the phenotype of root growth in *abo6* mutant, it indicates that ROS generated in root tissues have a negative regulatory effect on root growth and development (He et al., 2012; Yang et al., 2014). Other reports also show the same regulatory relationship, exogenous application of GSH promotes the growth of *Arabidopsis* roots, while reducing the content of related antioxidants will inhibit the development of roots (Sanchez-Fernandez et al., 1997; Vernoux et al., 2000). Study has found that the domesticated mung bean seedling is detected the high content of ABA and the hypersensitivity to ABA, and showing a faster stomatal closure response, enhancing extracellular ROS production, and elevating antioxidant enzyme activity to adapt to environmental stresses, such as the damage caused by the burst of oxidation generated by osmotic stress, that is beneficial to the growth and development of mung bean seedlings (Sahu and Kar, 2018). Although the molecular interaction mechanism of ABA, ROS and antioxidant in the process of plant domestication is not clear, it still shows that there is a possible cross network which regulates plant stress tolerance and growing development.

Plant roots are often exposed to a variety of abiotic stresses, among them, high salt stress is the most common adversity condition, which strongly restricts the growth of plant roots and acts as a stress signal to promote the production of root tissue ROS. In plants, overmuch concentration of ROS will be toxic to plant cells. Therefore, the level of ROS must be strictly controlled (Qi et al., 2017). In order to reduce the oxidative damage of ROS to cells and maintain the intracellular redox balance, plants have evolved many defense systems, including ROS enzyme scavenging systems, such as ascorbate peroxidase (APX), superoxide dismutase (SOD), catalase (CAT), and glutathione peroxidase (GPX) (Foyer and Noctor, 2005). A large number of studies have shown that the ROS enzyme scavenging system is related to the tolerance and growth status of plants under abiotic stress (Dong et al., 2013; Huda et al., 2013; You et al., 2014). Constitutive overexpression of *APX2* in the gain-of-function mutants can significantly enhance plant drought tolerance, improve plant water utilization efficiency, and maintain H_2_O_2_ homeostasis in plant cells, play a role in chloroplast protection and plant growing development (Rossel et al., 2006; Wu et al., 2018). Overexpression of *APX* also shows high tolerance to salt stress in transgenic plants (Lu et al., 2007; Yan et al., 2016). Similarly, constitutive overexpression of *OsGSTU4* (glutathione S-transferase) in *Arabidopsis* can increase plant tolerance to salt and oxidative stresses, thereby promoting plant growth and development under adversity conditions (Sharma et al., 2014).

Under abiotic stresses, plants will cause premature senility of leaf through the changes of endogenous factors, which are important limiting factors for plant growth and crop yields. In the processes of these endogenous factors regulate leaf premature senility, ABA plays an important role in the connection between the oxidative damage of cell and the signal molecule response to abiotic stresses. ABA signaling mediates the expression of *NYC*, *bZIP*, *WRKY* and other transcription factors on transcription level, and indirectly affects the premature senility of leaf. In addition, Ca^2+^ signaling, ROS generation, and protein degradation also lead to leaf premature senility (Asad et al., 2019). Both exogenous environmental stimulating factors and endogenous senility factors can induce the activation of plant antioxidant systems and promote NADPH oxidase to catalyze the generation of ROS (Jiang and Zhang, 2002; Foreman et al., 2003; Ma et al., 2012). At the same time, ABA-mediated ROS production by NADPH oxidase acts as a second messenger in the ABA signal transduction pathway, negatively regulating the speed of ABA signal transmission, and then inducing leaf senescence (Kwak, 2003). Reports have shown that exogenous ABA can activate the NOX activity of genes encoding *OsNox2*, *OsNox5*, *OsNox6*, *OsNox7*, etc. in rice, and then promote the generation of ROS in guard cells. (Jiang and Zhang, 2002; Zhang et al., 2009). Thereinto, the expression of *OsNox5* and *OsNox7* are dependent on low and high concentrations of ABA respectively, indicating that OsNox5 and OsNox7 have a significant correlation with the level of ABA in plant tissues (Li et al., 2018).

In the process of plant photosynthesis, excessive light damage will lead to the degradation of the photosynthetic reaction center binding protein D1 by increasing the level of ROS and reducing the activity of PSII. In the light-dependent leaf senescence process, ABA can promote the degradation of D1 protein to trigger light damage (Gan and Amasino, 1997), however, partial shielding of light will cause the accumulation of low concentration ABA in local leaves and delay the senescence of leaves (Wang et al., 2016), which indicate that low horizontal ROS accumulation can cause lower photoinhibition and oxidative damage, and induce the degradation of D1 protein. On the contrary, low concentration ABA can not only inhibit the degradation of D1 protein, but also accelerate the biosynthesis of D1 protein. Study has found that ABA can induce the expression of the D1 protein-encoding gene *PsbA* during leaf senescence, thereby increase the content of D1 protein (Asad et al., 2019), indicating that low concentration ABA and ROS have opposite effects on the regulation of leaf senescence. In the darkness, the accumulation of D1 protein in rice leaves can reduce the level of ABA and further promote the photodamage repair of the PSII system.

Studies have reported that the transcription factor OsMADS25 regulates the elongation of main root and the number of lateral roots in rice through mediating the ABA signal pathway and the ROS scavenging system. Meanwhile, OsMADS25 can specifically activate the expression of *OsP5CR*, a key element of proline synthesis, in addition, OsMADS25 can activate the expression of *OsYUC4* to promote auxin signaling, which in turn regulates root growth (Xu et al., 2018). In the process of plant root development, in addition to auxin and glucose, ROS also acts as a key signal molecule to regulate the activity of plant meristems. In particular, ROS is continuously generated in the root tip. The level of ROS controls the direction and scope of root tip growth (Liszkay et al., 2004). As a byproduct of plant cell metabolism, low level of ROS play a key role as a second messenger, regulating many important growth and development processes, including the division and differentiation of root tip meristem cells (Dunand et al., 2007; del Pozo, 2016). However, excessive ROS accumulation can cause oxidative damage to root tip cells. In mutants with altered ROS production or disrupted redox balance, the growth statuses of the taproots show significant differences (Dunand et al., 2007; Tsukagoshi et al., 2010; Yu et al., 2016; Zeng et al., 2017). The latest research showed that the increase of Ca^2+^ signal level in the cytoplasm caused by the external environment or growing development can indirectly inhibits the ABI4-mediated ABA signal pathway, thereby promoting the germination of *Arabidopsis* seeds (Kong et al., 2015). ROS also can promote the increase of Ca^2+^ content in the cytoplasm (Schroeder and Hagiwara, 1990; Pei et al., 2000; Kwak, 2003). Meanwhile, ABI4 can promote the generation of ROS through inhibiting the expression of *VTC2* (Yu et al., 2019), and ABA-induced H_2_O_2_ production to activate Ca^2+^ channels in the plasma membrane (Pei et al., 2000). Therefore, whether ROS becomes an intermediate between Ca^2+^ signal and ABA signal through feedback effect in this regulatory network remains to be studied.

## The roles of ABA and ROS in plant signaling

In plant cells, ABA is recognized and bound by ABA receptor RCAR/PYR1/PYL (Ma et al., 2009; Park et al., 2009), which interacts with downstream type 2C protein phosphatases (PP2Cs) to activate SNF1 (Sucrose-Non-Fermenting Kinase 1)-related protein kinase OPEN STOMATA1 (OST1)/SnRK2, which is inhibited by PP2C that promotes the dephosphorylation of Ser/Thr residues of OST1/SnRK2 to inactivate their activity (Umezawa et al., 2009; Mittler and Blumwald, 2015). Once activated, OST1 could be directly or indirectly combined, phosphorylate anion channel protein SLOW ANION CHANNEL-ASSOCIATED1, mediate ion release, cause guard cell movement, and promote stomatal closure (Geiger et al., 2009; Lee et al., 2009; Geiger et al, 2010; Brandt et al., 2012). Nicotinamide adenine dinucleotide phosphate (NADPH) oxidase is known as respiratory burst oxidase homologs (RBOHs), which is the main enzyme that promotes the generation of ROS in terrestrial plants (Umezawa et al., 2010; Mittler et al., 2011), and that is a homologue with mammalian NADPH oxidase in structure and function. RBOHs cross the plasma membrane to transport electrons from the inside to outside of the cell to form O^2.-^, and then generates H_2_O_2_ spontaneously or through superoxide dismutase (SOD) (Suzuki et al., 2011). At present, studies have reported that there are 10 RBOHs members in *Arabidopsis*, among them, RBOHD and RBOHF are the most representative members, and they play a very key role in the process of apoplast ROS synthesis, such as, caused by the interaction between plants and pathogens (Torres et al., 2002; Nühse et al., 2007; Zhang et al., 2007a). OST1 targets to bind RBOHF on the plasma membrane, phosphorylates the N-terminal Ser-13 and Ser-174 residues of RBOHF, and then generates H_2_O_2_ through the SOD outside the plasma membrane, whereas the regulation of ABA to NADPH through OST1 is ineffective in guard cells of the *ost1* knockout mutant, and then resulting in the hindrance of ROS production (Wang and Song, 2008; Sirichandra et al., 2009). Interestingly, H_2_O_2_ production through OST1 regulation can be used as a feedback signal to inactivate PP2C activity, thereby further promoting the activity of OST1, forming a positive feedback loop (Raghavendra et al., 2010).

While plants live in drought and high salt environments, osmotic stress can induce a large amount of endogenous ABA enrichment to regulate the expression of stress resistance genes and the physiological processes in cells, which plays a very important role in the response of plants to adversity stress (Shi et al., 2013; Xie et al., 2016). Research evidence shows that ROS as a second messenger is an indispensable signal molecule in the ABA signal transduction pathway (Kwak, 2003; He et al., 2012; Shi et al., 2013; Suzuki et al., 2013; Gong et al., 2020). ABA stimulates the production of ROS through the NADPH oxidase of guard cells, and then induces the closure of stomata to adapt to drought stress condition (Xie et al., 2016). However, the mechanism disappears that ABA induces H_2_O_2_ production and stomata closure in the *rbohD*/*rbohF* double mutant, and reduces the inhibitory effect of ABA on root growing development in *Arabidopsis* (Kwak, 2003). The downstream transcription factor ABI1 (ABA INSENSITIVE1, ABI1) of ABA signal transduction pathway forms a complex with phosphatidic acid, which interacts with RBOHD/F to stimulate the generation of ROS (Zhang et al., 2009). Nevertheless, ABA loses the ability to induce ROS generation in the guard cells of the phospholipase Da1 mutant (*PLDa1*) (Zhang et al., 2004). ABA insensitivity factor ABI2 (ABA INSENSITIVE2, ABI2) also can interact with GPX3 to regulate the redox balance in guard cells (Miao et al., 2006). Research findings have confirmed that ROS is an important second messenger in the ABA signal transduction pathway.

ABA induces the opening of Ca^2+^ channels caused by hyperpolarization in the cytoplasmic membrane of guard cells, thereby increasing the concentration of Ca^2+^ in the cytoplasm (Schroeder and Hagiwara, 1990; Pei et al., 2000). As a signal molecule, ROS, especially H_2_O_2_, plays a very important role in ABA-mediated activation of Ca^2+^ channels (Pei et al., 2000; Kwak, 2003). The increase of the Ca^2+^ concentration in the cytoplasm activates the anion channel which binding the plasma membrane, leading to the depolarization of the plasma membrane, resulting in inhibition of the KAT1 K^+^ channel (Osakabe et al., 2014). Interestingly, OST1 can phosphorylate the C-terminal domain of KAT1, thereby inhibiting K^+^ channel activity (Sato et al., 2009). Therefore, it is hypothesized that ABA and Ca^2+^ play a synergistic role in regulating K^+^ channel closure. Ca^2+^ is very important for the activation of RBOHD in the cell. The combination of Ca^2+^ and EF hand is necessary for the activation of RBOHD (Ogasawara et al., 2008). In addition, the activation of CPKs promoted by Ca^2+^ is also essential for the activation of RBOHD (Boudsocq et al., 2010), such as, CPK5 phosphorylates the N-terminus of RBOHD to promote its activity (Dubiella et al., 2013). Although Ca^2+^ and CPKs are upstream of RBOHD-induced ROS generation, ROS can promote intracellular Ca^2+^ accumulation and activate CPK5 in turn (Dubiella et al., 2013). Studies have reported that ROS and Ca^2+^ may play an important role in the signal transmission between cells, and the long-distance transmission of Ca^2+^ and ROS is called Ca^2+^ wave or ROS wave, which may be effective in the system response of plants to biotic or abiotic stress as an important signaling molecule (Mittler et al., 2011; Gilroy et al., 2016).

In the ABA signal transduction pathway, OST1 is an essential signal element in the process of ABA-induced ROS generation (Mustilli et al., 2002). Moreover, studies have found that OST1 can phosphorylate the N-terminus of RBOHF (Sirichandra et al., 2009), but it is unclear whether this phosphorylation is necessary for OST1-mediated signal transduction. The latest research shows that OST1 persulfidation can enhance ABA-induced intracytoplasmic Ca^2+^ signal transduction and stomatal closure, and this persulfidation is related to the two Cys residues of OST1, which are very close to the kinase catalytic region and phosphorylation site of OST1 (Chen et al., 2020). However, it is interesting that recent studies have found that the phosphorylation of OST1 by BAK1 (BRI1-associated receptor kinase1, BAK1) is necessary for the process of ABA-induced ROS generation in guard cells (Shang et al., 2016). Considering that BAK1 is a co-receptor for LRR-RLKs (leucine-rich repeats receptor-like kinases, LRR-RLKs), it is speculated that OST1 may also be regulated by a certain LRR-RLKs. Excitingly, the latest research has discovered for the first time that the plant H_2_O_2_ receptor HPCA1 is an LRR receptor kinase located on the plasma membrane of the cell with two special Cys residues in its extracellular domain. Because the sulfhydryl group on the Cys residue is the target of H_2_O_2_ oxidation, H_2_O_2_ will cause the oxidation of the extracellular Cys residues of HPCA1 in guard cells, thereby activating the intracellular kinase activity of HPCA1, and further triggering the activation of Ca^2+^ channels and causing Ca^2+^ internal flow, which subsequently causes the stomatal closure. HPCA1-mediated H_2_O_2_-induced activation of Ca^2+^ channels is necessary for stomatal closure in guard cells (Wu et al., 2020). This receptor kinase-mediated H_2_O_2_ sensing mechanism is not similar to any known H_2_O_2_ receptor or sensor protein reported in other organisms. However, whether the endogenous H_2_O_2_ regulates the Cys residue on OST1, and then regulates ABA signal pathway, or exists a feedback signal synergy pathway between ABA and H_2_O_2_, maybe that is a new research entry point. Meanwhile, studies have found that H_2_O_2_ promoted by ABA inhibits the interaction of PP45 and DMI3 in rice, thereby activating DMI3, which can combine with calmodulin induced by the ABA signal pathway to regulate the ABA signal transduction process (Ni et al., 2019). It can be seen that H_2_O_2_ regulates the ABA signaling pathway in different ways.

## The crosstalk between ABA and ROS in plant hormones network

**Figure 2:**
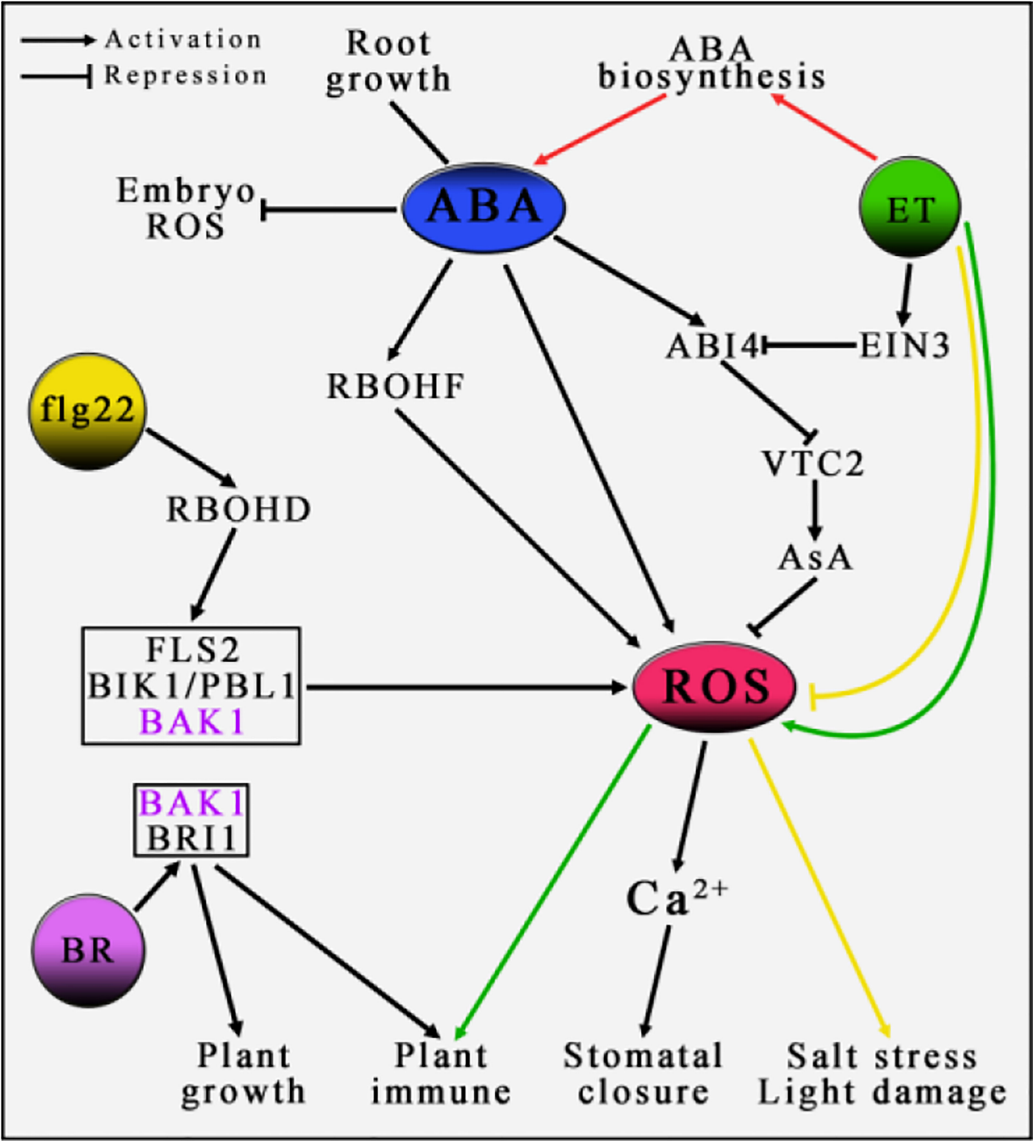
The role of ROS in the regulation of plant by hormones. In the process of plant growth and development, plant hormones play an important regulatory role, such as ABA, ET, BR, etc. In the entire regulatory network, ROS may play a central pivotal role. It remains to be elucidated whether BAK1, as an important element in the pathway of ROS generation induced by flg22, is the key site associated with the BR signaling pathway. Lines of the same color indicate the same regulatory pathway. For detailed explanation, please see text.

Plant hormones can bind and induce downstream regulatory factors to affect plant growth and development in biotic and abiotic stresses, such as ABA, ethylene and brassinolide. Among them, ABA and ethylene, as two vital plant hormones, play important roles in many aspects of plant growth and development, including the response to adversity stresses (Bleecker and Kende, 2000; Finkelstein et al., 2002; Vishwakarma et al., 2017; Yin et al., 2017). Reports have shown that the accumulation of ROS in plants can not only regulate ABA-induced stomata closure and the activity of root meristems mediated by salicylic acid, but also play an important role in the Na balance mechanism in buds regulated by ethylene (Pei et al., 2000; Jiang et al., 2012; Jiang and Bel, 2013; Xu et al., 2017a). Therefore, it is speculated that there may be an important regulatory relationship between ROS and the plant hormone network, which plays an important role in the growing development of plants (Xia et al., 2015). For example, ABA induces the accumulation of ROS, which leads to the increase of Ca^2+^ content in guard cells, regulates stomatal closure in turn (Pei et al., 2000; Wang and Song, 2008). On the contrary, exogenous ABA treatment will decrease the level of ROS in rice seed germination embryos (Ye et al., 2012) (**Figure 2**). Ethylene can trigger the accumulation of ROS in the ozone-induced PCD and response to pathogenic bacteria to achieve the primary response of plant immunity (Overmyer et al., 2000; Overmyer et al., 2003; Mersmann et al., 2010; Tintor et al., 2013). Recent studies have shown that ethylene can regulate the MPK pathway by promoting RBOHF-mediated accumulation of ROS, which in turn leads to the accumulation of NO in plants and promotes the closure of stomata, in addition, as an important factor in the MPK pathway, MPK3/6 plays a role in the upstream of EIN2, indicating that ROS directly participates in the signal transduction regulation of ethylene (Zhang et al., 2021). Conversely, ethylene replies to the seedlings’ response to salt stress and photooxidation damage by reducing the level of ROS (Zhong et al., 2009; Peng et al., 2014). The results of these studies indicate that there may be a mechanism by which ABA and ethylene co-regulate the accumulation of ROS, which is a key element in elucidating the regulatory network between plant hormones and ROS. Interestingly, many reports show that ABA and ethylene exhibit two relationships in different biological processes, synergy and antagonism (**Figure 2**). For example, ethylene inhibits the growth of rice roots by promoting the biosynthesis of ABA (Yin et al., 2015). In addition, when plants respond to nutrient stress, the endoderm of roots will form plugging, while the effects of ABA and ethylene on root plugging are completely opposite (Barberon et al., 2016). ABA and ethylene jointly regulate the histone acetylation of SWI-INDEPENDENT 3 LIKE1 and SWI-INDEPENDENT 3 LIKE2 to achieve an antagonistic effect on seed dormancy (Wang et al., 2013).

Ascorbate acid (AsA), as an important and highly effective non-enzymatic antioxidant, not only removes ROS generated during photosynthesis and growing development, but also plays an important role in the response of plants to biotic and abiotic stresses (Davey et al., 2000; Smirnoff and Wheeler, 2000; Gallie, 2013; Akram et al., 2017). With the discovery of multiple roles of AsA in the growing development of various plants, AsA and its regulatory mechanisms have received more and more attention (De Tullio and Arrigoni, 2004; Gallie, 2013; Bulley and Laing, 2016). Studies have reported that AsA plays an important role in the antagonistic relationship between ABA and ethylene. The AsA synthesis gene *VTC2* is antagonistically regulated by the downstream factors ABI4 and EIN3 in the ABA and ethylene signal pathway respectively. EIN3 directly binds to the promoter of *ABI4* to inhibit its expression, and ABI4 directly binds to the promoter of *VTC2* to inhibit its expression, revealing a new mechanism of ABA and ethylene antagonistic regulation, that is to say, regulating the biosynthesis of AsA through the transcriptional cascade antagonism of EIN3-ABI4-VTC2, thereby affecting the accumulation of ROS in *Arabidopsis thaliana* seedlings (Yu et al., 2019) (**Figure 2**). With continuous literature reports, the regulatory relationship between ABA and ethylene on plant growing development has shown a complex regulatory network. However, the detailed mechanisms and key connections are still poorly understood.

Similar to the pathway that ABA induces RBOHF to trigger apoplast ROS generation, triggering apoplast ROS generation by the activation of RBOHD induced by bacterial flagellin epitope flg22 (Chinchilla et al., 2006), and the combination of receptors on the plasma membrane is composed of FLS2 (FLAGELLIN-SENSING-2), BIK1 (BOTRYTIS-INDUCED KINASE1) and PBL1 (PBS1-Like 1) (Lu et al., 2010; Zhang et al., 2010). Among them, BIK1 and PBL1 are germane cytoplasmic protein kinases, and FLS2 is a leucine-rich repeat receptor kinases (LRR-RLKs). When flg22 induces apoplast ROS generation, another LRR-RLK, BAK1 (BRI1-ASSOCIATED RECEPTOR KINASE 1) acts as a co-receptor to sense flg22 signal and jointly promote the transphosphorylation of FLS2, BAK1 and BIK1/PBL1 (Chinchilla et al., 2007; Heese et al., 2007; Lu et al., 2010; Zhang et al., 2010), thereby triggering a transient burst of Ca^2+^ in the cytoplasm (Li et al., 2014; Ranf et al., 2014; Monaghan et al., 2015), apoplast ROS generation (Lu et al., 2010; Zhang et al., 2010), and resistance to bacteria and pathogenic fungi (Veronese et al., 2006; Zhang et al., 2010). Interestingly, BAK1 acts as a brassinolide (BR) signaling regulatory element interacts with BR receptor BRI1 (Li et al., 2002; Nam and Li, 2002; Russinova et al., 2004), and co-phosphorylated to perceive and transmit BR signals (Wang et al., 2005; Wang et al., 2008), thereby mediate BR-regulated plant growth and development. Studies have found that BAK1 is not necessary in the FLS2-mediated plant immune signal pathway and BR-induced plant PAMP signal to pathogenic fungi, indicating that BAK1 may not be an intermediate element in the ABA, ROS and BR signaling networks (**Figure 2**). However, BAK1 can phosphorylate SnRK2.6 (Shang et al., 2016), and BIN2 kinase interacts with SnRK2.2 (Belin et al., 2006). It remains to be proved that SnRK2s and BIN2 may link ABA and BR signaling pathways, and mediate ROS generation as the key element.

## Future perspective

**Figure 3:**
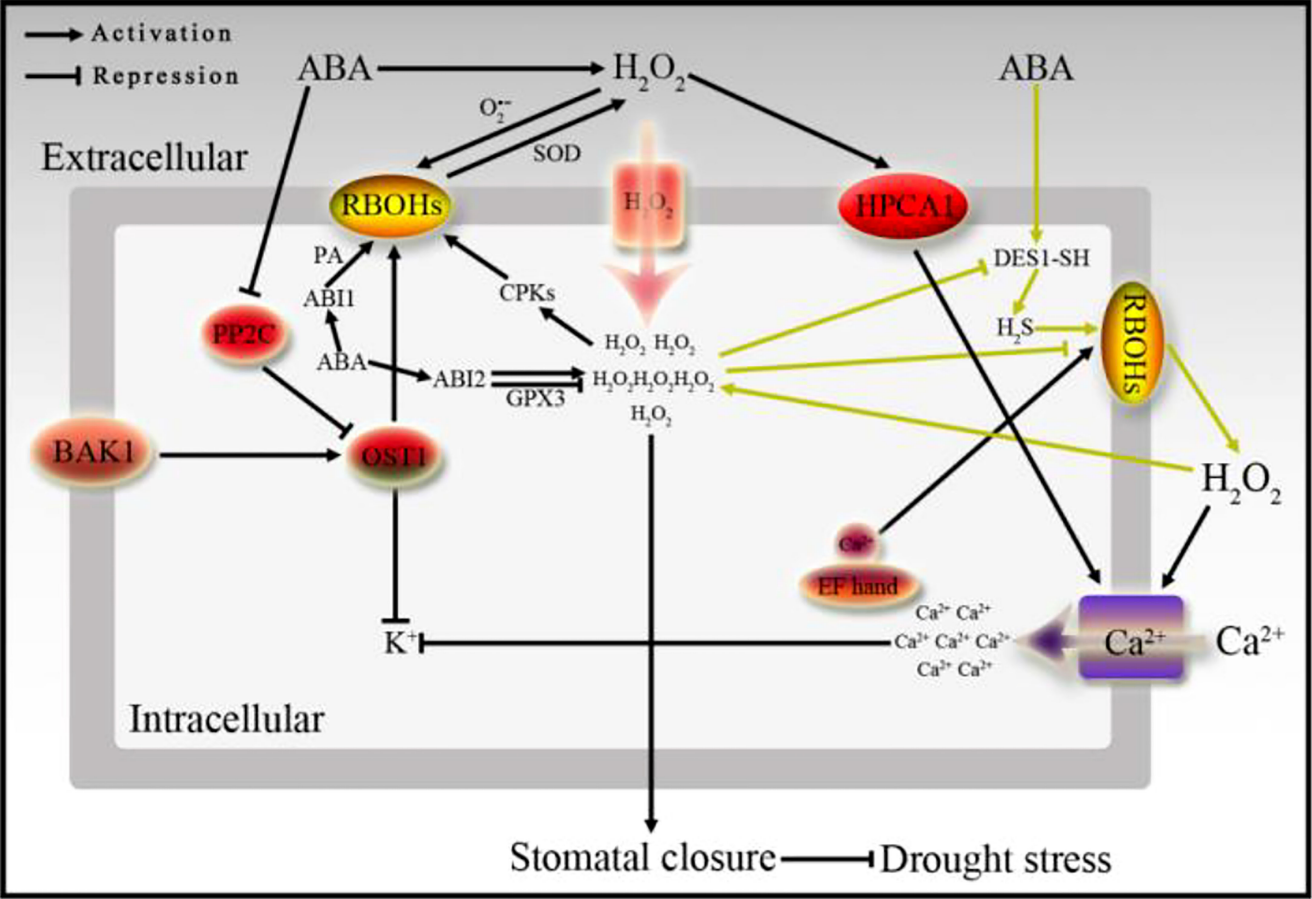
The relationship between ABA signaling and H_2_O_2_ in stomatal movement. ABA signal is transmitted downward through PP2C and OST1, or ABI1 and ABI2, meanwhile, ABA can promote the generation of H_2_O_2_ induced by RBOHs on the plasma membrane, and forms a microcirculation by H_2_S. H_2_O_2_ is sensed by the H_2_O_2_ receptor HPCA1, and the H_2_O_2_ signal is transmitted into the cell to promote Ca^2+^ influx, inhibit K^+^ accumulation, and then causes stomata to close.

Under normal physiological conditions, the production and consumption of ROS in cells is in a dynamic equilibrium state. An appropriate amount of ROS is of great significance to the signal transduction in plant cells and the immunity of pathogenic microorganisms. However, excessive ROS will lead to oxidative stresses, and the generation of stresses will be accompanied by a series of negative effects, such as irreversible damage to proteins, biofilms, DNA and RNA. Recent study has found that increased ROS can cause DNA breakage in maize sperm cells, resulting in haploid maize plants (Jiang et al., 2022). There are many factors that cause plant cells to produce excessive ROS, such as ABA, high light, adversity stress, and pathogen infiltration. Among them, apoplast ROS is mainly induced by the plasma membrane binding protein NADPH oxidase (RBOH). In addition to the NADPH oxidase of apoplasts, there are also some oxidases located on the plasma membrane of chloroplasts, peroxisomes, and mitochondria, which are also related to the production of ROS, especially during the interaction between plants and pathogens (Bolwell and Wojtaszek, 1997; Apel and Hirt, 2004). The main elements involved in the generation of apoplast ROS are rich in leucine and belong to the LRR-RLK class of protein molecules. The newly discovered plant H_2_O_2_ receptor HPCA1 is also an LRR-RLK protein molecule located on the cytomembrane. Cys residues exposed outside the cytomembrane are covalent modification by H_2_O_2_, leading to HPCA1 autophosphorylation of the part inside cell, and then gets activated (Wu et al., 2020). H_2_O_2_ is an important molecule for cells to respond to external stresses and internal signals. H_2_O_2_ enters into the cell through the water channel membrane proteins and covalently modified cytoplasmic proteins to regulate signal transduction and cellular processes (**Figure 3**).

It has been reported in the literature that H_2_S is involved in ABA-regulated ROS production and stomatal closure, conversely, excessive accumulation of ROS inhibits H_2_S production, thus forming a mini-regulatory circuit (Jie et al., 2020) (**Figure 3**). In addition, hydrogen sulfide (H_2_S) modifies SnRK2.6/OST1 through *S*-nitrosylation to participate in the molecular mechanism of regulating ABA signaling to induce plant stomatal closure (Chen et al., 2020). Interestingly, two S-sulfhydryl modification sites-Cys residues are found in SnRK2.6, when these two Cys residues are modified by S-sulfhydryl, the activity of SnRK2.6 and its interaction with transcription factors downstream of ABA signaling all will be promoted (Chen et al., 2020). Meanwhile, ABA and H_2_S-induced stomatal closure and Ca^2+^ influx will be less sensitive, and leading to water loss and decreased drought resistance in the *ost1-3* mutant (Chen et al., 2020). In addition, ABA induces the DES1 enzyme in the guard cells to catalyze the production of H_2_S, in turn, the H_2_S positively regulates ABA signal transduction through the persulfidation of SnRK2.6 to affect calcium influx, and finally regulates stomatal movement, therefore, a new ABA signal transduction mechanism is proposed that relies on the regulation of sulfhydryl modification (Chen et al., 2020) (**Figure 3**). As mentioned above, the N-terminus of RBOHs contain multiple regulatory sites or motifs, including phosphorylation sites, EF hand, and phosphatidic acid binding elements, etc, which make RBOHs with binding and regulating effects (Ogasawara et al., 2008) (**Figure 3**). In addition to phosphorylation and sulfhydrylation, ubiquitination modification which is generally modified by E3 ubiquitin ligases is also an important regulation of ABA perception, signal transduction and response. PYR/PRLs, PP2Cs, PP2As, transcription factors and proteins encoded by ABA response genes are all targets of E3 ubiquitin ligase-mediated ubiquitination modification, and will be further degraded by the 26S proteasome. At present, it has been confirmed that a variety of E3 ubiquitin ligases are related to ABA signaling, including CRLs, RING and U-box types of ubiquitin ligases (Yu et al., 2015), and another type of E6-AP ubiquitin ligases have not been reported to be related to ABA signaling. Many ABA-related factors affect ABA signal transduction and stress resistance through the regulation of E3 ubiquitin ligases in plant growing, for example, ABA synthesis elements ABA1 and ABA2 (Lee et al., 2010), ABA receptor molecules PYL1 and PYL4 (Bueso et al., 2014), PYL8 (Irigoyen et al., 2014), ABA signaling factor ABI1 (Park et al., 2009; Kong et al., 2015), ABI2 and HAB2 (Wu et al., 2016), ABA signaling downstream transcription factor ABI3 (Kurup et al., 2000), ABI5 (Smalle et al., 2003; Lee et al., 2010; Seo et al., 2014), ABF1 (Chen et al., 2013), ABF3 (Chen et al., 2013), ATH86 (Lechner et al., 2011), SDIRIP1 (Zhang et al., 2007b; Zhang et al., 2015), and ABA downstream response gene *RD21* (Kim and Kim, 2013), etc. In addition, recent studies have reported that the new E2 ubiquitin coupling enzyme UBC27 and the RING type E3 ubiquitin ligase AIRP3 form a specific E2-E3 complex and activate the ubiquitin ligase activity of AIRP3. Meanwhile, both UBC27 and AIRP3 can directly interact with the ABA co-receptor ABI1 to regulate ABA signaling and plant drought stress response (Pan et al., 2020). Ubiquitination modification basically regulates all the related fields of ABA in plants, and further affects plant growth and development, stress resistance, and interaction with pathogens. Although the modification and regulation mechanisms of many ABA-related elements have been studied and reported, the detailed ABA signaling network still needs to be verified and supplemented.

The photosynthesis of plants will be stimulated by high light to generate ROS in the cells, thereby causing light damage to plant cells. The latest research has found that the phytochrome-related transcription factor PHYTOCHROME-INTERACTING FACTORS (PIFs) directly binds to the promoter of the *ABI5* and activating the expression of *ABI5* specifically to regulate the ABA signaling pathway in the dark, meanwhile, ABA receptors PYL8/PYL9 can directly interact with PIFs and mediate this transcriptional regulation of *ABI5* (Qi et al., 2020). PIFs are the key factors that integrate multiple signal pathways to regulate plant growth and development, and widely involved in a variety of plant hormones, such as gibberellin, ethylene, auxin, brassinolide, etc. meanwhile, mediated by external environmental factors, such as high temperature, high light, etc. in the signal regulation network (Paik et al., 2017). Whether the ROS generated by high light is involved in the signal transduction network that regulated by these plant hormones, we believe that the discovery of more ROS functions will play a very important role in the improvement of the signal regulation network. In addition, the interaction between ABA and auxin has been proven, ABA can promote auxin-mediated plant growth inhibition (Tiryaki and Staswick, 2002; Monroe-Augustus et al., 2003; Belin et al., 2009). In turn, auxin can promote ABA-mediated inhibition of seed germination and leaf senescence under different oxidative stress conditions (Liu et al., 2007; Mahmood et al., 2016). It shows that ABA and auxin co-promote the process of seed germination and seedling growth (Belin et al., 2009; Tsukagoshi et al., 2010). It is interesting to report that ABA can make use of ROS from mitochondria to antagonize the regulation of auxin in *Arabidopsis*. In turn, auxin can also attenuate the inhibitory effect of ABA on seed germination (He et al., 2012), indicating that there is both synergy and antagonism between ABA and auxin, which may be related to the concentration of plant hormones, and ROS plays an important role therein. In terms of plant defenses against pathogens, ABA may coordinate or antagonize the SA, JA, ET and other signal pathways through direct or indirect mechanisms, thereby regulating the antibacterial properties of plants (Mohr and Cahill, 2007; Robert-Seilaniantz et al., 2007; Derksen et al., 2013). This seems to be regarded as the main mechanism by which ABA induces the sensitivity of plants to a variety of pathogens. However, the role of ROS in these mechanisms has not been fully elucidated, this is also future research hotspots in plant disease resistance.

Studies have found that just restoring the expression of *RBOHD* in the xylem parenchyma or phloem cells in the *rbohD* mutant can restore the systemic ROS signal in plant. The transcriptional expression and the adaptive mechanism cause by systemic stress response to deal with the high light stimulation applied to a single leaf, indicating that RBOHD plays a key role in regulating the generation of the vascular bundles ROS, inducing systemic signal transduction and adaptation in *Arabidopsis*. Meanwhile, the signal integration of ROS and calcium, which are important signaling molecules for long-distance transport between cells or in plants, and electricity and hydraulic pressure occurs in the vascular bundle along with the process of systemic signal transduction (Zandalinas et al., 2020) (**Figure 3**). A recent review article also reported that ROS is produced, enriched, converted and transported across the membrane in several organelles of plant cells, including chloroplasts, mitochondria, endoplasmic reticulum, peroxisomes and vacuoles, etc. Meanwhile, ROS is transported across the membrane between plant cells to achieve ROS signal transmission throughout the plant (Mittler et al., 2022). This study suggests that we can express RBOHD/F specifically in different tissues, cells and organelles to perfect the understanding of RBOHD/F in mediating ROS fluctuations, and understanding of plant systemic responses induced by ROS. Improving plant ABA and ROS signal regulation components and pathways will help to further understand how plants adjust their endogenous signals and regulation strategies according to environmental changes, so as to obtain better survival ability in the natural. Additionally, that will provide new insights for the elucidation of plant hormone signal network and regulatory mechanism.

## Author contributions

SHL, SL and LW designed this research and wrote the manuscript. LW, QZ, MC and MZ contributed to the provided guidance of the whole manuscript. LW, SL, QZ and RW reviewed and revised the manuscript. SHL, and LW prepared the figures. NL, SW, YC and LZ checked and revised the language and format of the manuscript. All authors contributed to the article and approved the submitted version.

## Funding

This study were funded by Hebei Natural Science Foundation (C2022402021, C2022402026, C2019402343, C2019402430); The Science and Technology Research Project of University in Hebei Province (QN2020205); Science and Technology Research and Development Plan Project of Handan (19422011008–49, 21422012331).

## Conflict of interest

The authors declare that the research was conducted in the absence of any commercial or financial relationships that could be construed as a potential conflict of interest.

## Publisher’s note

All claims expressed in this article are solely those of the authors and do not necessarily represent those of their affiliated organizations, or those of the publisher, the editors and the reviewers. Any product that may be evaluated in this article, or claim that may be made by its manufacturer, is not guaranteed or endorsed by the publisher.
